# 540 Burn-Specific Triage Guidelines in State-Based Crisis Standards of Care

**DOI:** 10.1093/jbcr/irac012.168

**Published:** 2022-03-23

**Authors:** Lori Chrisco, Felicia Williams, Booker King, Rabia Nizamani

**Affiliations:** North Carolina Jaycee Burn Center, Chapel Hill, North Carolina; UNC Jaycee Burn Center, Chapel Hill, North Carolina; UNC Jaycee Burn Center, Chapel Hill, North Carolina; UNC Jaycee Burn Center, Chapel Hill, North Carolina

## Abstract

**Introduction:**

In times of crisis, medical institutions must utilize contingency plans to ensure the highest quality of patient care. When these plans are overwhelmed, crisis standards of care may be adopted, resulting in modifications in resource allocation. The current coronavirus pandemic has created tremendous strains on hospitals throughout the world, with periodic shortages in equipment, PPE, ICU beds, and personnel. These pressures have been great enough at times to result in several states implementing crisis standards of care to allow hospitals to triage patients and "do the most good possible for the largest number of people with limited resources". However, these guidelines may not account for the unique needs of burn patients, whose care is often resource intensive. We examined state-based crisis standards of care guidelines in the United States to ascertain the degree to which triage of burn patients was addressed.

**Methods:**

Internet search engines were used to locate state-specific actionable “crisis standards of care” or “scarce resource allocation” policies available before October 1, 2021. Once identified, these guidelines were further examined to determine whether explicit information was provided to direct the triage of burn patients.

**Results:**

Of the 50 states and the District of Columbia, only 35 states (70%) were confirmed to have official crisis standards of care policies that could be implemented by healthcare institutions during the current pandemic. Additionally, guidelines from non-government entities were identified for 4 states (Florida Bioethics Network, Ohio Hospital Association, Missouri Hospital Association, and West Virginia Hospital Association). Of the 39 plans available, only 13 (26%) provided specific information regarding triage of burn patients during implementation of crisis standards of care.

**Conclusions:**

Crisis standards of care are heterogenous throughout the United States and have varying levels of specificity. The majority of states and the District of Columbia do not provide clear, actionable guidance on the triage of burn patients.

